# The resting cyst of dinoflagellate *Scrippsiella acuminata* host bacterial microbiomes with more diverse trophic strategies under conditions typically observed in marine sediments

**DOI:** 10.3389/fmicb.2024.1407459

**Published:** 2024-07-22

**Authors:** Yunyan Deng, Fengting Li, Lixia Shang, Zhangxi Hu, Caixia Yue, Ying Zhong Tang

**Affiliations:** ^1^CAS Key Laboratory of Marine Ecology and Environmental Sciences, Institute of Oceanology, Chinese Academy of Sciences, Qingdao, China; ^2^Laboratory for Marine Ecology and Environmental Science, Qingdao Marine Science and Technology Center, Qingdao, China; ^3^Center for Ocean Mega-Science, Chinese Academy of Sciences, Qingdao, China; ^4^College of Fisheries, Guangdong Ocean University, Zhanjiang, China

**Keywords:** acetogenic bacteria (acetogen), ammonium-oxidizing (anammox) bacteria, anaerobic respiration, chemotrophic bacteria, dinoflagellate resting cysts, nitrate-reducing bacteria (NRB), selective preservation, sulfate-reducing bacteria (SRB)

## Abstract

Variation in the condition of marine sediments provides selective preservation milieus, which act as a key determinant for the abundance and distribution of dinoflagellate resting cysts in natural sediments. Microbial degradation is an understudied biological factor of potential importance in the processes. However, gaps remain in our knowledge about the fundamental information of the bacterial consortia associated with dinoflagellate resting cysts both in laboratory cultures and in the field. Here we used *Scrippsiella acuminata* as a representative of cyst-producing dinoflagellates to delineate the diversity and composition of bacterial microbiomes co-existing with the laboratory-cultured resting cysts, and to explore possible impacts of low temperature, darkness, and anoxia (the mock conditions commonly observed in marine sediments) on the associated bacterial consortia. Bacterial microbiome with high diversity were revealed associated with *S. acuminata* at resting stage. The mock conditions could significantly shift bacterial community structure and exert notably inhibitory effects on growth-promoting bacteria. Resting cysts under conditions typically observed in marine sediments fostered bacterial microbiomes with more diverse trophic strategies, characteristic of prominently enriched anaerobic chemotrophic bacteria generating energy via respiration with several different terminal electron acceptors, which yielded more acidic milieu unfavorable for the preservation of calcareous resting cysts. Our findings suggest that there is complex and dynamic interaction between dinoflagellates resting cysts and the associated bacterial consortia in natural sediments. This intrinsic interaction may influence the maintenance and/or accumulation of dinoflagellate resting cysts with potential of germination and initiation blooms in the field.

## 1 Introduction

Nature contains an abundance of resting stages. The production of resting stages is currently known to be a common life cycle trait for hundreds of coastal plankton organisms belonging to various realms (Ellegaard and Ribeiro, 2018; Belmonte and Rubino, 2019). This non-motile stage can either germinate near the sediment surface to provide the inoculum for subsequent population or, be buried by sediment deposits over time and entrained into the sedimentary record. Buried resting stages can be resuspended into the water column by mixing events (e.g., storms) or other disturbances (e.g., dredging) (Belmonte and Rubino, 2019). The resting stage of dinoflagellates is well-known as resting cysts, representing the benthic, dormant stage in life cycle of dinoflagellates (Bravo and Figueroa, 2014; Tang et al., 2021). Although it is not clear how long dinoflagellates resting cysts can survive in natural sediments while still be capable of germinating given favorable conditions, they may remain alive and maintain viability for more than 100 years (Lundholm et al., 2011; Ribeiro et al., 2011; Delebecq et al., 2020). One of the reasons for resting cysts drawing extensive scientific attentions is that dinoflagellates are the greatest agents of global harmful algal blooms (HABs) events (Smayda, 1997), comprising about 40% of all HABs-forming species (Jeong et al., 2021). Resting cysts are highly important in the ecology of dinoflagellates, particularly for those HABs-causative members, as they are involved in seeding, initiation and termination of HABs, as well as genetic recombination to potentially increase the adaptation, resistance to unfavorable environmental conditions, geographic expansion of populations via natural and anthropological activities, and the provision of possible indicators for alien species invasion (see reviewed in Bravo and Figueroa, 2014; Tang et al., 2021).

The abundance and distribution of resting cysts in the field are influenced by multiple factors (Belmonte and Rubino, 2019; Persson and Smith, 2022). Encystment is considered to be an adaptive response to external stresses, such as temperature, salinity, daylight length, and nutrient depletion (see reviewed in Bravo and Figueroa, 2014; Tang et al., 2016). Therefore, these environmental fluctuations affect the natural cysts production and their spatial distribution (Belmonte and Rubino, 2019; Liu et al., 2023). Meanwhile, in the sediment, physical–chemical degradation influence cyst preservation, also driving reconstruction of cysts assemblage (Zonneveld et al., 2010, 2019; Gray et al., 2017; Rodríguez-Villegas et al., 2021, 2022). Sedimentary records of dinoflagellates are highly selective, preserving only for resistant resting cysts, and may be biased under specific circumstances (Persson and Smith, 2022; Dale, 2023). This likely explains some discrepancy and gaps existing between the past resting cyst records and the current dinoflagellate monitoring data (Anderson et al., 2014; Díaz et al., 2018; Jung et al., 2018; Kremp et al., 2018). Several sediment physical–chemical characteristics have been put forward to account for selective preservation of dinoflagellate cysts, such as temperature, dissolved oxygen, pH, sediment texture, and redox potential (Zonneveld et al., 2010, 2019; Gray et al., 2017; Rodríguez-Villegas et al., 2021, 2022; Persson and Smith, 2022).

Microbial degradation is an understudied biological factor of potential importance influencing resting cysts preservation in natural sediments (Persson, 2000). Bacteria utilize organic matter derived from oceanic primary production by varied strategies, including attacking on dead and living resting stages by using hydrolytic enzymes (Smith et al., 1992, 1995; Zonneveld et al., 2010; Belmonte and Rubino, 2019). Generally, compared with vegetative cells, resting cysts having thick or multi-layered cyst walls are more resistant to microbial degradation (Bravo and Figueroa, 2014; Tang et al., 2016). However, it should be noted that not all resting cysts of dinoflagellates can be viewed as resistant to all digestion and/or degradation (Dale, 2023). At least, the thin “non-fossilizable” cysts of many naked dinoflagellates are vulnerable to bacterial degradation (Tang and Gobler, 2012a; Dungca-Santos et al., 2019). Indeed, it is not difficult to find broken and damaged resting cysts in the field of plankton research, even for “fossilizable” species, such as *Lingulodinium polyedrum* (Persson, 2000; Belmonte and Rubino, 2019). The population maintenance of *Scrippsiella* resting cysts has been documented to be threatened by bacterial degradation, particularly in eutrophic areas (Shin et al., 2013, 2014), suggesting that differences in bacterial degradation between the environments may be important in the resting cysts preservation (Persson, 2000). Nonetheless, gap remains in our knowledge about the fundamental information (e.g., species diversity, community composition) of the bacterial consortia associated with dinoflagellate resting cysts both in laboratory cultures and in the field.

The thecate dinoflagellate *Scrippsiella acuminata* (formerly *S. trochoidea*; Kretschmann et al., 2015) is a cosmopolitan, HABs-forming, and toxic species (Tang and Gobler, 2012b). Due to more readily producing resting cysts, it has been adopted as representative species for studies on dinoflagellates life history (Deng et al., 2017; Guo et al., 2021; Li et al., 2021, 2022; Wang et al., 2022; Wu et al., 2022; Yue et al., 2022). In the present study, *S. acuminata* was chosen as a representative dinoflagellate to characterize the diversity and composition of bacterial microbiomes co-existing with the laboratory-cultured resting cysts and preliminarily explore the possible impact of low temperature, darkness, and anoxia (the conditions typically observed in marine sediments) on the whole bacterial consortia. Our findings raise the possibility that there are still complex and dynamic interactions between dinoflagellates at resting stage and associated bacterial microbiomes in natural sediments, which might potentially affect cysts preservation in the field.

## 2 Materials and methods

### 2.1 Culture maintenance and resting cysts preparation

The strain IOCAS-St-1 of *Scrippsiella acuminata* initially established from the Yellow Sea of China was obtained from the Marine Biological Culture Collection Centre, Institute of Oceanology, Chinese Academy of Sciences (IOCAS) and regularly maintained in our laboratory at IOCAS. Vegetative cells used for routine maintenance were kept in f/2-Si medium (Guillard, 1975) based on pre-filtered (0.22 μm membrane filter, Millipore, Billerica, MA, USA) and autoclaved (121°C for 30 min) natural seawater (salinity of 32–33). A mixed penicillin–streptomycin solution (100×, Solarbio, Beijing, China) with final concentration of 3% was added into the medium immediately before inoculation. Cultures were incubated at 20 ± 1°C in an incubator with a 12:12 h light: dark cycle, illuminated by a bank of cool white fluorescent lights providing a photon flux of ~100 μmol photons m^−2^ s^−1^.

Resting cysts were produced according to Yue et al. (2022) and briefed as follows. Vegetative cells cultured in 6-well culture plates (Corning, US; 10 mL in each well) were used for resting cysts production, which were incubated at abovementioned routine maintenance conditions except for adding f/2-Si medium with one-thousandth dilution of N and P nutrients (Yue et al., 2022). Cyst formation was checked every other day under inverted microscope (Olympus IX73). For the species, *S. acuminata*, there are clear distinctions in morphological features between vegetative cells and resting cysts (Deng et al., 2017), which allowed a swift judgement via light microscopy between the two types of cells. Resting cysts were general harvested from the cultures that had been inoculated for more than 30 days. Vegetative cells with their two flagella swam in the plates whereas resting cysts without flagellum settled at the bottom. Therefore, it was not difficult to physically separate them. The obtained resting cysts were washed several times with sterile filtered seawater until no motile cells (vegetative cells or planozygote) observed in the samples under microscope. The long duration of culturing and the washing steps made the vast majority of the harvested cells (>99%) were cysts as observed under a light microscope before further treatment.

### 2.2 Samples treatment and collection

For vegetative cell samples, vegetative cells (initial density of ~2 × 10^3^ cells mL^−1^) inoculated into 300 mL medium in 500 mL Pyrex flask were cultured at the normal condition used for routine maintenance. The day of inoculation was recorded as Day 0. Samples were harvested on Day 5 (at exponential growth stage and labeled as “V1”) and Day 10 (at stationary growth stage and labeled as “V2”), respectively. For resting cyst samples, the harvested newly formed resting cysts were labeled as sample “C0” (Table 1). Resting cysts stored under conditions typically observed in marine sediments (Table 1) were performed referring to the previous studies (Li et al., 2021, 2022) and briefed as follows. A total of 8 treatment combinations (Table 1) were applied in the current study. The darkness treatments were conducted by wrapping the cyst-containing tubes with aluminum foils before exposure to different storage temperatures. Low temperature treatment was performed in either an incubator (15°C) or a refrigerator (4°C without light). The anoxia environment was achieved by ventilating the cyst-containing tubes with nitrogen gas and monitoring the dissolved oxygen levels of the samples with an oxygen meter (PreSens Microx 4, PreSens, Regensburg, Germany). The Na-resazurin indicator (0.1% *w/v*) was also added into culture medium to ensure anoxia conditions via color change (Xiao et al., 2010). Three samples of V1, V2 and C0 were categorized as “VC0” group, while the rest 8 samples were classified as “C” group (Table 1). All cells in each sample (~10^5^ cells) were pelleted in a 1.5 mL centrifuge tube and immediately used for genomic DNA extraction.

**Table 1:** Samples treatments in the current study.

Grouping	Sample ID	Temperature	Oxygen	Light	Storage period	Remarks
VC0	V1	21°C	Yes	Yes	Not applicable	Vegetative cells at exponential growth stage
VC0	V2	21°C	Yes	Yes	Not applicable	Vegetative cells at stationary growth stage
VC0	C0	21°C	Yes	Yes	Not applicable	Newly formed resting cysts
C	C1L1Y021	21°C	No	Yes	1 month	Resting cysts stored under conditions typically observed in marine sediments
C	C1L0Y021	21°C	No	No	1 month	
C	C2L0Y021	21°C	No	No	2 month	
C	C3L0Y021	21°C	No	No	3 month	
C	C3L0Y015	15°C	No	No	3 month	
C	C1L0Y04	4°C	No	No	1 month	
C	C2L0Y04	4°C	No	No	2 month	
C	C3L0Y04	4°C	No	No	3 month	

### 2.3 Genomic DNA extraction, PCR amplification, and high-throughput sequencing

DNA extraction was conducted using a Plant DNA Extraction Kit (Tiangen, Beijing, China) and eluted with 60 μL TE buffer. Nuclear-free water processed through DNA extraction served as the negative control. The bacterial 16S rDNA V3-V4 hypervariable regions were amplified using the primer 341F (5’-CCTACGGGNGGCWGCAG-3’) and the primer 805R (5’-GACTACHVGGGTATCTAATCC-3’) (Logue et al., 2016). PCR reactions were performed in a 25 μL mixture containing 50 ng of template DNA as described in Deng et al. (2023). Nuclease-free water was used as the sample blank. PCR cycling conditions included an initial denaturation at 98°C for 30 s, followed by 35 cycles of denaturation at 98°C for 10 s, annealing at 54°C for 30 s, elongation at 72°C for 45 s, and a final extension at 72°C for 10 min. Amplicons were monitored on a 2% agarose gels, followed by purification with Gel Extraction Kit (Axygen Biosciences, Union City, CA, USA). Retrieved DNA were pooled for sequencing on the Illumina NovaSeq platform (LC-Bio Technology Company, Hangzhou, Zhejiang, China) with 250 bp paired-end reads.

### 2.4 Bioinformatic and statistical analyses

The raw data were deposited to NCBI BioProject database under the number PRJNA1065589^1^. Quality trimming and length filtering on raw reads were performed in Fqtrim (version 0.9.7^2^), and chimeric reads were further filtered in Vsearch (version 2.3.4) (Rognes et al., 2016). Paired-end reads were assigned to samples based on their unique barcode, trimmed of barcodes and primer sequences, and merged with FLASH tool (version 1.2.6) (Magoč and Salzberg, 2011). The amplicon sequence variants (ASVs, sequences clustered at 100% sequence similarity) were obtained with DADA2 package (version 3.6.1) (Callahan et al., 2016). All the ASVs were annotated by conducting BLAST search against the SILVA database (Quast et al., 2013) and denominated at different taxonomic levels. Relative abundance of each ASV was estimated based on its read counts normalized to the total number of good quality reads. Alpha diversity indices (Simpson evenness, Shannon diversity, Chao1 richness, Observed species richness, and Goods coverage) were calculated via QIIME (Quantitative Insights Into Microbial Ecology) (version 2) (Bolyen et al., 2019) to analyze the complexity of species diversity. Beta diversity analyses of PCA (Principal component analysis) and PCoA (Principal coordinated analysis) were performed to compare community composition between the two groups. Both alpha and beta diversity analyses were conducted at the ASVs level. Venn diagrams showing the shared and unique features were plotted with BioVenn^3^. The significance of variance was tested with one-way ANOVA or *t*-test using the software SPSS (version 22.0) (SAS Institute Inc., Cary, NC, USA). The significance level in all statistical analyses was set at 0.05 unless otherwise stated. The functional potential of bacterial microbiome was predicted using PICRUSt (Phylogenetic Investigation of Communities by Reconstruction of Unobserved States) algorithm (version 2.3.0-b) to make inferences from KEGG database. The inputs to PICRUSt follow standard formats which produced from the16S rRNA gene-based microbial species compositions analysis done in QIIME scripts (Bolyen et al., 2019). The differentially abundant gene families and pathways were assessed using the software STAMP (version 2.1.3) (Parks et al., 2014) subjected to t-test, the threshold of *p* < 0.05 was used to display functions with statistically significant difference (at confidence interval 95%).

## 3 Results

### 3.1 Global overview of bacterial microbiomes co-existing with the laboratory-cultured *Scrippsiella acuminata*

**Figure 1:**
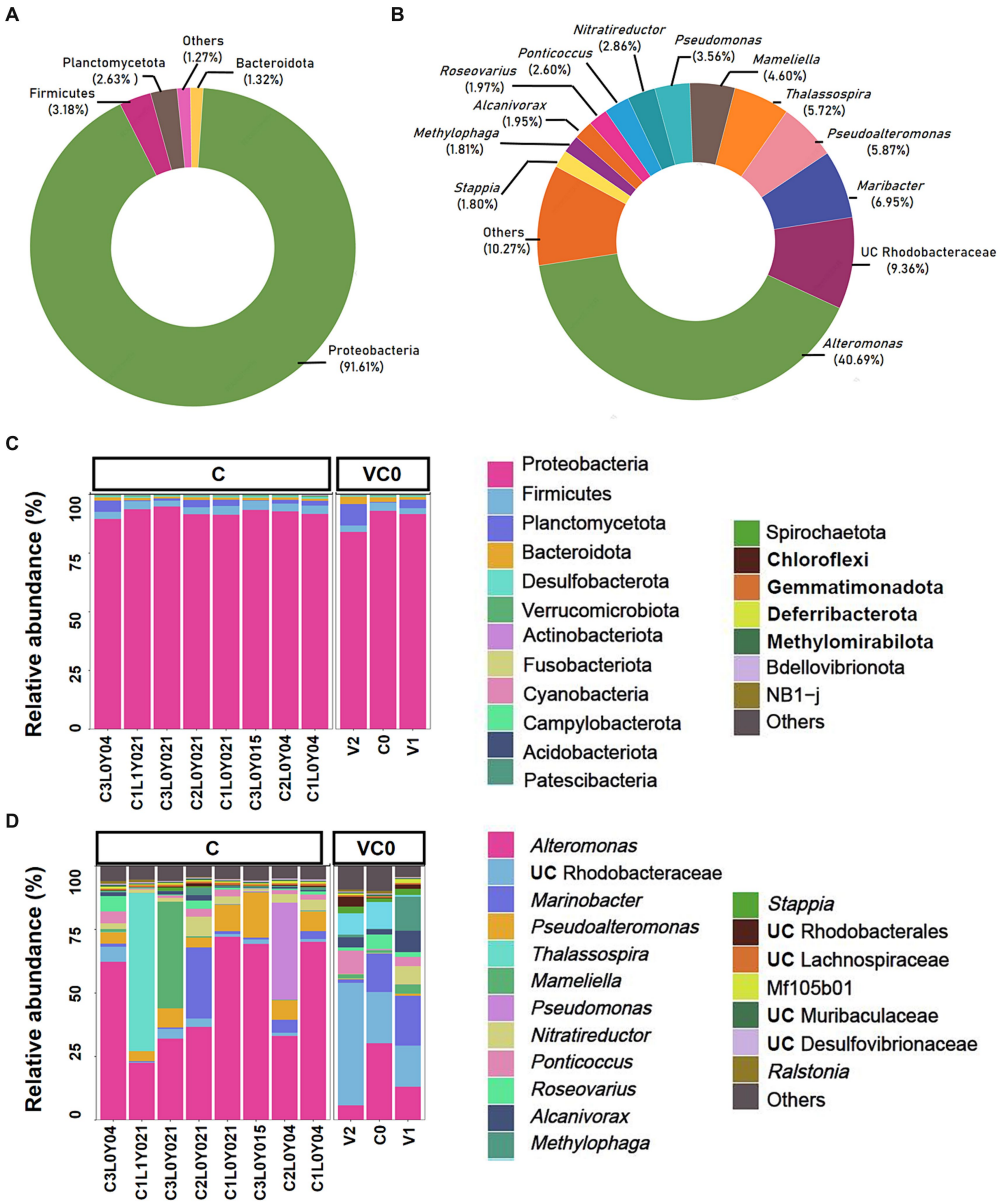
Taxonomic composition of the whole bacterial microbiome **(A,B)** and the relative abundance (%) of bacteria communities present in the 11 samples **(C,D)**. **(A)** At phylum level; **(B)** at genus level; **(C)** the top 20 phylum; **(D)** the top 20 genera. Pie charts show relative abundances of the phyla/genera for the entire bacterial community. The *X*-axis shows sample IDs. The *Y*-axis shows the relative abundance (%) in total effective ASVs. UC, unclassified.

The prokaryotic 16S rRNA gene metabarcoding sequencing generated 926,751 raw reads, corresponding to 0.45 Gb of raw data. The raw sequencing data were submitted to National Center for Biotechnology Information (NCBI) under the BioProject number PRJNA1065589. A total of 755,555 clean reads were obtained, with effective sequences per sample varied from 65,173 to 73,314 (Supplementary Table S1). Good’s coverage values of all samples were 1.00 (Supplementary Table S2) and rarefaction curves tended to reach saturation with increasing amounts (Supplementary Figure S1), suggesting that sufficient sequences were harvested to uncover the vast majority of prokaryotic taxa. Dereplication using DADA2 pipeline yielded 1,490 qualified amplicon sequencing variants (ASVs, sequences clustered at a 100% sequence identity), among which 1,478 ASVs belonging to bacteria kingdom were further assigned to 25 phyla, 60 classes, 124 orders, 222 families, 435 genera, and 543 species via blasting against SILVA database (Supplementary Table S3). The entire bacterial assemblage was mainly composed of Proteobacteria (91.61%), Firmicutes (3.18%), Planctomycetota (2.63%), and Bacteroidota (1.32%) (Figure 1). At genus level, *Alteromonas* (40.69%), UC (unclassified) Rhodobacteraceae (9.36%), *Maribacter* (6.95%), *Pseudoalteromonas* (5.87%), *Thalassospira* (5.72%), *Mameliella* (4.60%), *Pseudomonas* (3.56%), *Nitratireductor* (2.86%), *Ponticoccus* (2.60%), *Roseovarius* (1.97%), *Alcanivorax* (1.95%), *Methylophaga* (1.81%), and *Stappia* (1.80%) were the most dominant genera (relative abundance >1%), which together contributed up to 89.73% of the whole bacterial microbiomes co-existing with the laboratory-cultured *S. acuminata* (Figure 1).

### 3.2 Comparative analysis of ASV diversity and community composition of bacterial microbiomes between VC0 and C groups

**Figure 2:**
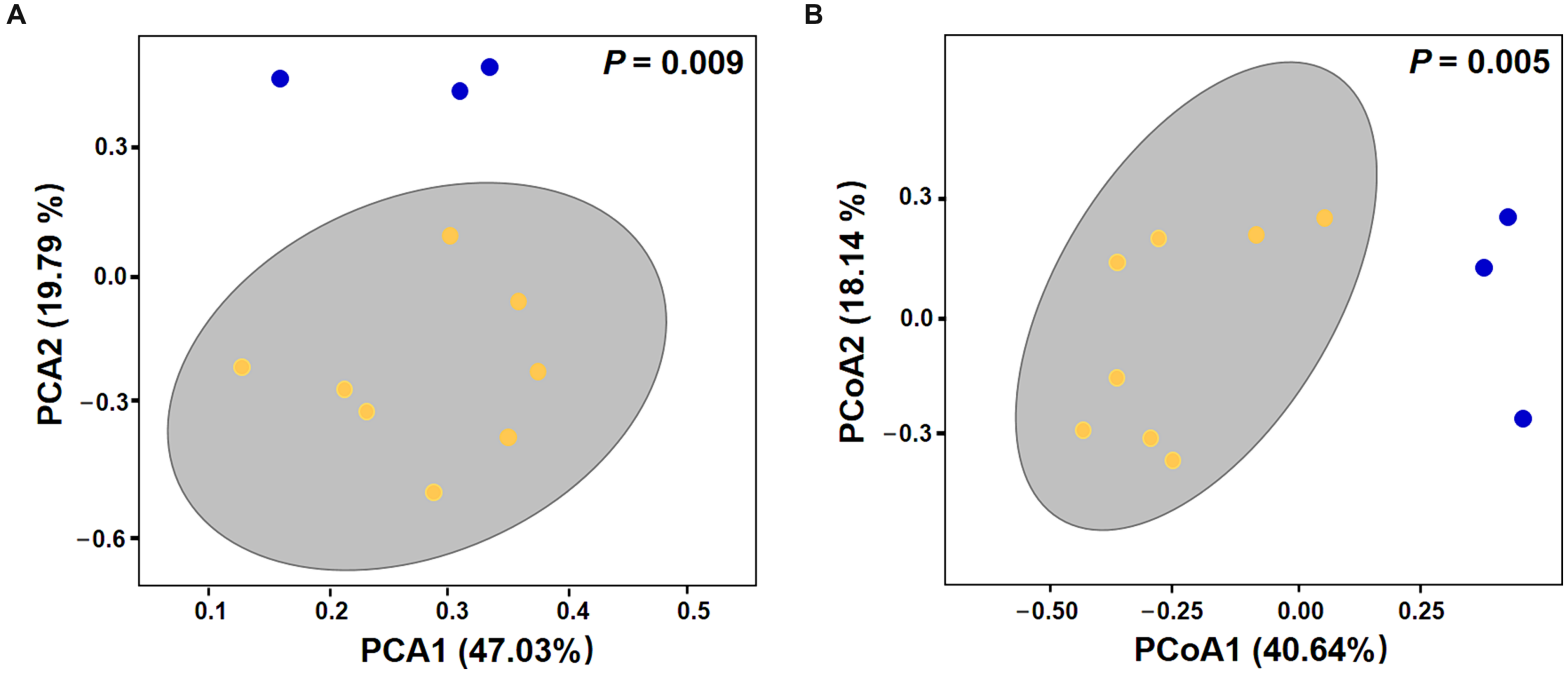
The beta diversity analysis of C (yellow) and VC0 (blue) groups. **(A)** Principal component analysis (PCA) performed at ASV level using QIIME (version 2) plugin. **(B)** Principal coordinate analysis (PCoA) based on Bray-Curtis distances at ASV level.

**Figure 3:**
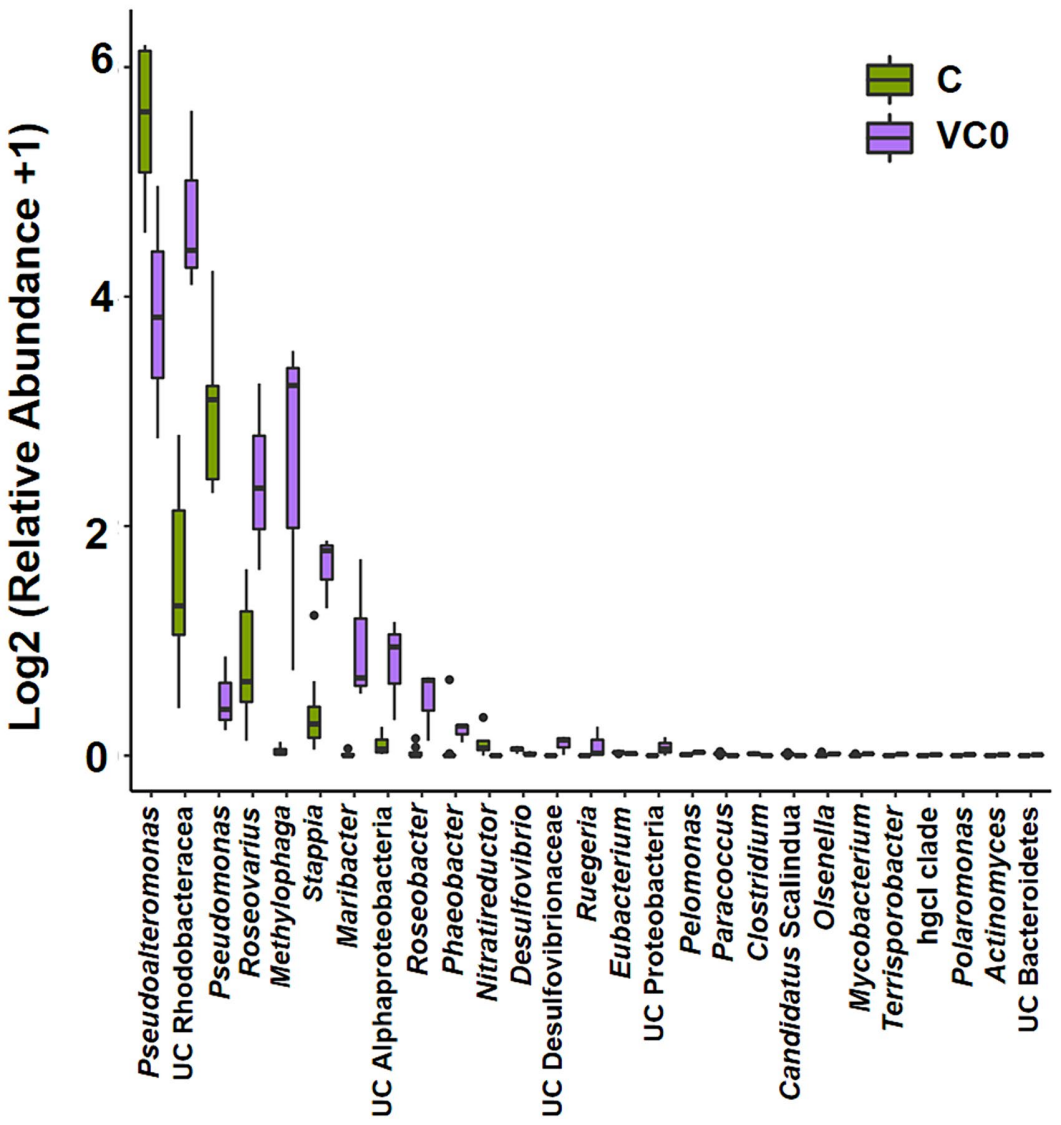
Bar plot of the 27 bacterial genera showing significantly differential abundance between C and VC0 groups. The *Y*-axis shows the relative abundance of these genera. The analysis were performed at the genus level for 16S rRNA gene amplicons via QIIME (version 2) plugin. Dot denotes outlier.

Overall, there was no significant difference in alpha diversity indices between VC0 (vegetative cells from which the resting cysts were initially produced and newly formed resting cysts exposed to normal condition used for routine maintenance) and C (resting cysts exposure to low temperature, darkness, and anoxia for different durations) groups (ANOVA, *p* > 0.05; Supplementary Figure S2a). In Beta diversity analyses, both PCA and PCoA plots showed that all the samples in C group formed a cluster distinct from VC0 group, whereas the 3 VC0 samples exhibited indiscernible affiliation with one another (Figure 2). Overall, the two groups shared 18 common phyla, while VC0 and C groups harbored 3 and 4 unique phyla, respectively (Supplementary Figure S2b). Significantly higher abundance of Firmicutes and lower abundance of Bacteroidota were found in C group than those in VC0 group (Supplementary Figure S3). At genus level, 231 genera were shared, while 59 and 143 unique genera were present in VC0 and C groups, respectively (Supplementary Figure S2c). A total of 27 genera showed significantly differential abundance between the two groups (Figure 3). Compared with VC0 group, C group had significantly lower abundances of *Roseovarius*, UC Rhodobacteraceae, *Methylophaga*, *Stappia*, *Maribacter*, UC Alphaproteobacteria, *Roseobacter*, *Phaeobacter*, *Ruegeria*, UC Proteobacteria, *Pelomonas*, *Olsenella*, *Mycobacterium*, *Terrisporobacter*, hgcI clade, *Polaromonas*, *Actinomyces*, UC Bacteroidetes, but greatly enriched in *Pseudoalteromonas*, *Pseudomonas*, *Nitratireductor*, *Desulfovibrio*, UC Desulfovibrionaceae, *Eubacterium*, *Paracoccus*, *Candidatus* Scalindua, *Clostridium* (Figure 3).

### 3.3 Comparative analysis of predicted metabolic inferences of bacterial microbiomes between VC0 and C groups

**Figure 4:**
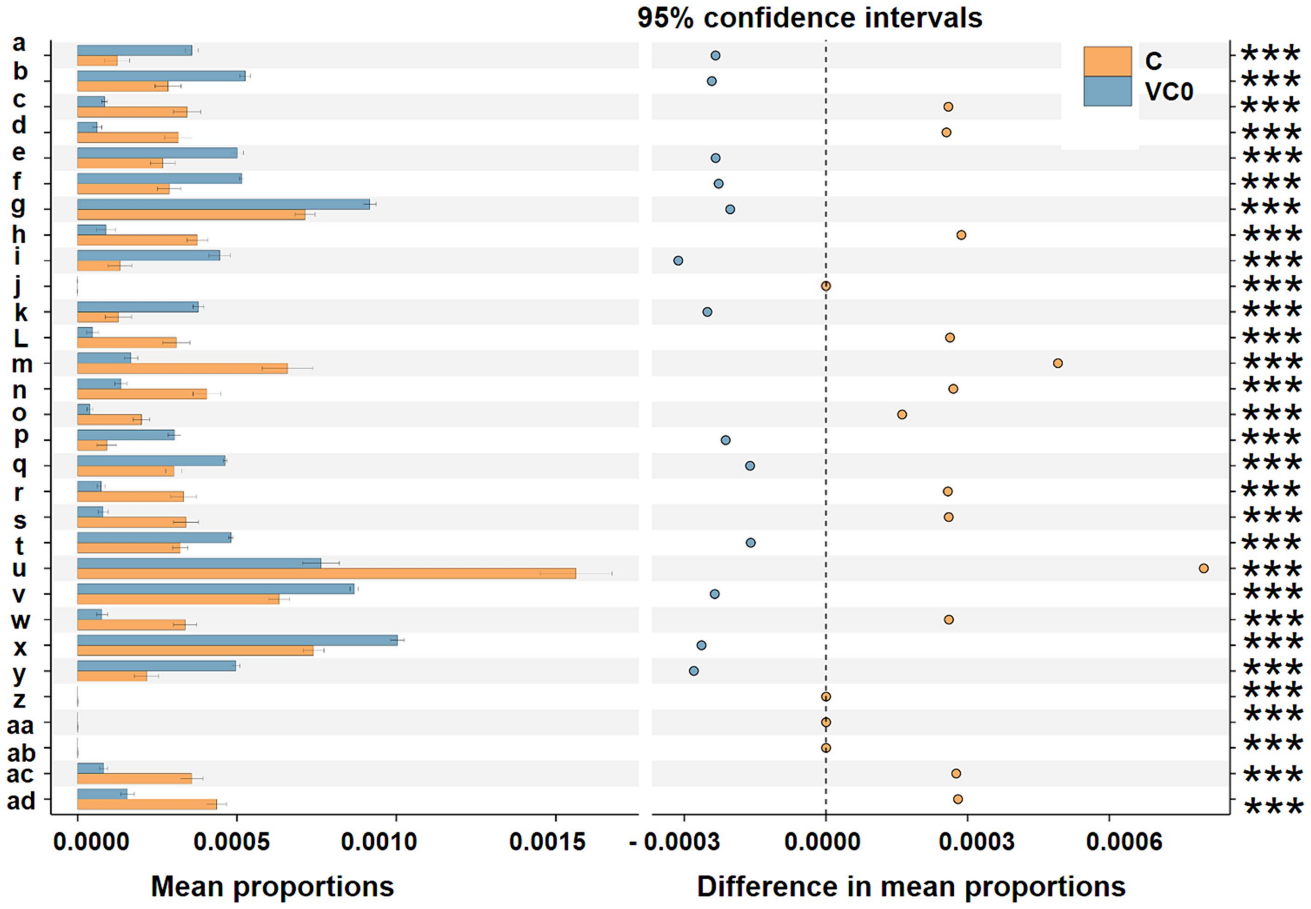
Prediction of the differentially functional inferences of bacterial communities between the C (orange) and VC0 (blue haze) groups in KO (KEGG Orthology) assignments. Gene functions were predicted from 16S rRNA gene-based microbial compositions at the ASV level using the PICRUSt algorithm to make inferences from KEGG annotated databases via QIIME (version 2) plugin. Relative signal intensity was normalized by the number of the genes for each indicated metabolic pathway. The 95% confidence interval is where 95% of the samples fall within this range. The right longitudinal axis are the *p*-values calculated by the *t*-test method. Symbols *** notes the difference at significant levels with *p* < 0.001. Mean proportions indicate the mean proportional of the functional term in the two group. Difference in mean proportions is shown as the distance from the dots to the dashed line, representing different values between the relative abundance of a certain functional term in a group and the mean proportions of the certain functional term. The dots of the two groups are on each side of the dashed line, in which group the relative abundance is higher, the dots will be shown as the color of the group. a: tRNA 2-selenouridine synthase [EC:2.9.1.-]; b: hydroxymethylpyrimidine [EC:2.7.1.49 2.7.4.7]; c: N-acetylmuramic acid 6-phosphate etherase [EC:4.2.1.126]; d: fumarylacetoacetate (FAA) hydrolase [EC:3.7.1.2]; e: hypoxanthine phosphoribosyltransferase [EC:2.4.2.8]; f: phosphocarrier protein; g: phosphate transport system substrate-binding protein; h: glucosamine kinase [EC:2.7.1.8]; i: phosphomannomutase/phosphoglucomutase [EC:5.4.2.8 5.4.2.2]; j: nitrate/nitrite response regulatory protein; k: uncharacterized protein; L: lipid A ethanolaminephosphotransferase [EC:2.7.8.43]; m: 2-dehydro-3-deoxygluconokinase [EC:2.7.1.45]; n: membrane dipeptidase [EC:3.4.13.19]; o: LysR family transcriptional regulator; p: urea carboxylase [EC:6.3.4.6]; q: ferredoxin; r: GLS; glutaminase [EC:3.5.1.2]; s: 1-aminocyclopropane-1-carboxylate deaminase [EC:3.5.99.7]; t: cell division protein ZapA; u: flagellin; v: arsenate reductase [EC:1.20.4.1]; w: fructuronate reductase [EC:1.1.1.57]; x: pyrC; dihydroorotase [EC:3.5.2.3]; y: Rrf2 family transcriptional regulator; z: adenylylsulfate reductase, subunit A [EC:1.8.99.2]; aa: dissimilatory sulfite reductase alpha subunit [EC:1.8.99.5]; ab: electron transport complex protein RnfG; ac: beta-glucosidase [EC:3.2.1.21]; ad: N-acetylglucosamine-6-phosphate deacetylase [EC:3.5.1.25].

To explore the ecological functions of the bacterial microbiomes between the two groups, their metabolic potentials were predicted using the PICRUSt (Phylogenetic Investigation of Communities by Reconstruction of Unobserved States) algorithm. The KEGG database was used as the basis for functional predictions in our study. For the top reference hierarchy (KEGG Level 1), genetic information processing, organismal systems, cellular processes, and unclassified process were dramatically over-represented in C group, whereas metabolism process showed significantly higher activity in VC0 group (Supplementary Figure S4a). For the sub-process level (KEGG Level 2), 11 functional modules (biosynthesis of other secondary metabolites, folding, sorting and degradation, cell motility, metabolism, transport and catabolism, cellular processes and signaling, signaling molecules and interaction, enzyme families, transcription, environmental adaptation, replication and repair) were markedly enriched in C group. While VC0 group had significantly higher abundances of 3 categories relevant to lipid metabolism, amino acid metabolism, metabolism of terpenoids and polyketides (Supplementary Figure S4a). In addition, predominant enrichment of 17 KO (KEGG Orthology) entries (*N*-acetylmuramic acid 6-phosphate etherase, fumarylacetoacetate hydrolase, glucosamine kinase, nitrate/nitrite response regulatory protein, lipid A ethanolaminephosphotransferase, 2-dehydro-3-deoxygluconokinase, membrane dipeptidase, LysR family transcriptional regulator, glutaminase, 1-aminocyclopropane-1-carboxylate deaminase, flagellin, fructuronate reductase, adenylylsulfate reductase subunit A, dissimilatory sulfite reductase alpha subunit, electron transport complex protein RnfG, beta-glucosidase, N-acetylglucosamine-6-phosphate deacetylase) were revealed in C group (Figure 4).

## 4 Discussion

### 4.1 Bacterial microbiomes with high diversity were revealed associated with *Scrippsiella acuminata* at resting stage

Although some case studies have reported that certain lineage of bacterial isolates might act as stimulatory or inhibitory “biotic factor” to affect cyst formation of some dinoflagellates (*Alexandrium* and *Lingulodinium*) (Adachi et al., 1999, 2002, 2003; Mayali et al., 2007), gap remains in current knowledge about global snapshot of bacterial associates with dinoflagellate resting cysts host. In contrast to the conventional notion of resting stage as an inactive metabolism stage (Ellegaard and Ribeiro, 2018; Wu et al., 2022), bacterial microbiomes of high diversity were recovered in the phycosphere of *S. acuminata* at resting stage. Since the clonal culture of *S. acuminata* used in this study has been maintained under routine laboratory conditions for more than 10 years, in which the well-adapted microbial communities have been established and maintained over long-term period. In general, compared with the extremelyly high degree of microbiodiversity found in terrestrial plant rhizospheres, less diversity of bacterial communities were commonly observed from phycospheres (the aquatic analog of the rhizosphere; Bell and Mitchell, 1972) surrounding microalgae (Krohn-Molt et al., 2017), especially for laboratory-cultured microalgae (Buchan et al., 2014; Seymour et al., 2017). Typically, less than 30 bacterial taxa at the species level were affiliated with microalgae-bacteria consortia (Krohn-Molt et al., 2017 and the references therein). In our study, the number of recovered bacterial genera in the 9 cyst samples varied from 90 to 199 (mean = 169), suggesting that the relatively higher diversity of bacterial consortia could reside and colonize in the laboratory-raised resting cysts under given conditions. It is likely that the relatively constant maintenance conditions in laboratory (e.g., temperature, nutrients supply, etc.) played roles in shaping and establishing bacteriome in our study. It would be very interesting to investigate the shift of natural microbial consortia coexisted with dinoflagellate resting cysts to laboratory raised phycospheres under different cultivation conditions.

### 4.2 The typical conditions commonly observed in marine sediments significantly influenced the bacterial community structure and exerted notably inhibitory effects on growth-promoting bacteria

In this study, the entire bacterial microbiome associated with *S. acuminata* was generally in accordance with the majority of previous records on bacterial assemblages recovered from laboratory cultures of dinoflagellates, in which *α*- and *γ*-proteobacteria are the predominant players (Deng et al., 2022 and the references therein). To explore the potential impact of low temperature, darkness, and anoxia (the mock conditions commonly observed in marine sediments) on the bacterial associates, we compared the shift in bacterial communities between VC0 and C groups. There was no significant difference found in terms of alpha diversity indices, indicating that the ASVs diversity was statistically similar between the two groups. However, beta diversity analysis showed that all the 8 samples in C group clustered together and were obviously distinguished from VC0 group, implying that C group exhibited conserved community structure and were significantly distinct from those of VC0 group. Hence, the typical conditions commonly observed in marine sediments could largely affect community composition rather than ASVs diversity of the bacterial microbiomes associated with resting cysts of *S. acuminata*.

Comparative analysis revealed that 18 genera had significantly less abundant in C group. Apart from 4 members (UC Rhodobacteraceae, UC Alphaproteobacteria, UC Proteobacteria, and UC Bacteroidetes) that have yet to be classified at the genus level, 10 of the other 14 bacteria have been previously reported as growth-promoting bacteria (GPB) which can support growth and development and/or improve other aspects of physiology and metabolism for algae and/or higher plants. The *Roseovarius* sp. and *Maribacter* sp. were established to induce growth and development of the green seaweed *Ulva* via production of phytohormone-like algal growth and morphogenesis-promoting factors (Li et al., 2023; Wichard, 2023). *Stappia* sp. were reported to ameliorate biomass and lipid productivity of marine microalga *Tetraselmis striata* in co-cultivation system (Park et al., 2017). *Roseobacter* and *Phaeobacter* were thought to promote algal growth by production of antibiotics and growth stimulants (Seyedsayamdost et al., 2011; Sauvage et al., 2022). *Ruegeria* was shown to produce tropodithietic acid as an antibiotic agent to enhance growth and health of microalgae by killing potential pathogens (Porsby et al., 2008). *Actinomyces* could produce anti-microbial agents to increase plant disease resistance (Lin and Xu, 2013). *Mycobacterium* and *Pelomonas* could facilitate growth of terrestrial plant *Doritaenopsis* and aquatic plant *Lemna minor*, respectively, by production of phytohormone indole-3-acetic acid (IAA) (Pan et al., 2020; Makino et al., 2022). *Polaromonas* was known to have growth-promoting effect on the higher plant *Beta vulgaris* (Okazaki et al., 2021). All these abovementioned bacteria were found to confer algae and/or plants beneficial effects. Plausibly, the notably decreased numbers of beneficial bacteria in C group suggested that the environmental changes were unfavorable for survival of these beneficial bacteria and/or a reduced need for beneficial functions from associated microbiome of resting cysts under the typical conditions commonly observed in marine sediments.

### 4.3 Resting cyst of *Scrippsiella acuminata* foster bacterial microbiomes with more diverse trophic strategies under conditions typically observed in marine sediments, characteristic by prominently enriched anaerobic chemotrophic members generating energy via respiration with several different terminal electron acceptors

A total of 9 bacterial genera (*Pseudoalteromonas*, *Pseudomonas*, *Nitratireductor*, *Desulfovibrio*, UC Desulfovibrionaceae, *Eubacterium*, *Paracoccus*, *Candidatus* Scalindua, *Clostridium*) with diverse metabolic processes coupling of energy yield exhibited greatly higher abundance in C group. *Pseudoalteromonas* is a group of facultative anaerobic taxa which were frequently detected from a range of extreme environments, including cold habitats and deep-sea sediments (Tang et al., 2020). They could develop peculiar mechanisms coping with extreme conditions and thus to thrive in these ecological niches (Parrilli et al., 2021). Some microbes could mediate oxidation–reduction (redox) reactions associated with metabolic processes coupling with inorganic element transformations to energy release in processes known as “dissimilatory transformations” (Lovley and Coates, 2000; Burgin et al., 2011). Different electron acceptors yield differing amounts of energy in redox reactions (Burgin et al., 2011). In our study, the significantly enriched genera in C group included bacteria capable of anaerobic chemotrophic metabolism linked to dissimilatory transformations using electron acceptors of nitrate, nitrite, sulfate, and carbon dioxide. *Pseudomonas*, *Nitratireductor*, *Paracoccus* are well-known nitrate-reducing bacteria which make use of nitrate as an alternative electron acceptor to oxygen during anaerobic nitrate respiration (Knowles, 1982; Baker et al., 1998; Labbé et al., 2004). Anaerobic ammonium oxidation (anammox) is a chemoautotrophic biological process in which ammonium is oxidized to nitrogen gas with nitrite as the electron acceptor under anoxic conditions (Schmid et al., 2007; Borin et al., 2013). *Candidatus* Scalindua is considered to be the dominant anammox genus in the marine environment (Borin et al., 2013). Supportively, differential function prediction at KO assignments indicated the entry annotated as “nitrate/nitrite response regulatory protein,” a component of bacterial two-component nitrate-sensing system (NarX-NarL), was predominantly higher in C group. The NarX-NarL is well-established to participate in regulation of nitrate and nitrite reductase synthesis, the key enzymes in anaerobic nitrate/nitrite respiration (Nohno et al., 1989). The dissimilatory sulfate reducing bacteria (SRB) use sulfate as the terminal electron acceptor in the oxidation of hydrogen and various organic compounds (Castro et al., 2000). *Desulfovibrio*, gram-negative mesophilic SRB, is one of the dominant SRBs in the marine environment (Castro et al., 2000). Desulfovibrionaceae represents a family comprises strict anaerobes with respiratory and fermentative types of metabolism. The vast majority members in Desulfovibrionaceae use sulfate as terminal electron acceptor (Galushko and Kuever, 2015). Furthermore, we found predominant enrichment of 2 KOs pertaining to sulfate respiration in C group than those in VCO group, including “adenylylsulfate reductase, subunit A” (Lampreia et al., 1994) and “dissimilatory sulfite reductase alpha subunit” (Wagner et al., 1998). Acetogenic bacteria (acetogens) are an anaerobic group that make a living from acetate formation from two molecules of CO_2_ via the Wood-Ljungdahl pathway (WLP) (Drake et al., 2008). Two acetogens, *Clostridium* and *Eubacterium* (Drake et al., 2008), as well as the KO entry of “electron transport complex protein RnfG,” a proton-translocating ferredoxin: NAD^+^ oxidoreductase coupling with ATP synthesis in multiple acetogens (Schuchmann and Müller, 2014), were found to be significantly enriched in C group. Collectively, our results revealed that more diverse trophic strategies of bacterial taxa associated with resting cysts of *S. acuminata* under conditions typically observed in marine sediments, characteristic of prominently enriched anaerobic chemotrophic bacteria which generated energy via respiration with several different terminal electron acceptors. It might be the metabolic adaptation of the bacteriome for more efficient exploitation of available resources when it comes to harsh environmental conditions thus to sustain population and gain more survival advantages.

The substantial increase of anaerobic respiration under mock sediment conditions likely yields more organic acids surrounding dinoflagellate host (Persson and Smith, 2022 and the references therein). This can affect resting cysts with an outer calcareous wall, such as *Scrippsiella* species, since calcium carbonate is prone to dissolve at low pH. The naked-type cyst of *S. acuminata*, cyst without calcareous wall, was observed in the natural surface sediments (Wang et al., 2007; Ishikawa et al., 2019) and proposed as a biological indicator for indicating hypoxia in coastal waters (Shin et al., 2013; Ishikawa et al., 2019). The inner organic wall of *Scrippsiella* is too delicate to be preservable (Lewis, 1988), and the naked-type cysts seem to be easily degraded in the natural sediments (Shin et al., 2013). Therefore, the more acidic milieu caused by bacterial activities might influence cyst population maintenance of *S. acuminata* and thus to reduce cyst density (Shin et al., 2013, 2018).

Our work provided the first global snapshots of the specific association/interaction of bacterial communities with host dinoflagellate resting cysts. Contrasting to the conventional notion of resting stage as an inactive physiological state, bacterial communities of high diversity were recovered from the phycosphere of *S. acuminata* resting cysts. The mock conditions commonly observed in marine sediments (low temperature, darkness, and anoxia) significantly affected bacterial community structure and yielded bacterial microbiomes with more diverse trophic strategies, characteristic of prominently enriched anaerobic chemotrophic bacteria generating energy via respiration with several different terminal electron acceptors. Our findings raise the possibility that there are still complex and dynamic interactions between dinoflagellates at resting stage and co-existing bacterial consortia. Further works on microbial communities associated with more cyst-producing dinoflagellate species both from natural populations in the field and in laboratory raised phycospheres is thus highly necessitated to comprehensively explore the potential influence of microbiome on the maintenance and/or accumulation of dinoflagellate resting cysts.

## Data availability statement

The datasets presented in this study can be found in online repositories. The names of the repository/repositories and accession number(s) can be found at: https://www.ncbi.nlm.nih.gov/, PRJNA1065589.

## Author contributions

YD: Conceptualization, Formal analysis, Funding acquisition, Investigation, Methodology, Software, Supervision, Visualization, Writing – original draft, Writing – review & editing. FL: Conceptualization, Data curation, Formal analysis, Funding acquisition, Investigation, Methodology, Software, Validation, Visualization, Writing – original draft. LS: Data curation, Formal analysis, Methodology, Validation, Writing – original draft. ZH: Conceptualization, Data curation, Formal analysis, Methodology, Software, Validation, Visualization, Writing – original draft. CY: Conceptualization, Data curation, Investigation, Methodology, Software, Validation, Visualization, Writing – original draft. YT: Data curation, Formal analysis, Funding acquisition, Project administration, Resources, Supervision, Validation, Visualization, Writing – review & editing.

## Footnotes

^1^ http://www.ncbi.nlm.nih.gov/bioproject/1065589

^2^ https://ccb.jhu.edu/software/fqtrim/index.shtml

^3^ http://www.biovenn.nl/index.php
