# Supplementary material for: The resting cyst of dinoflagellate Scrippsiella acuminata host bacterial microbiomes with more diverse trophic strategies under conditions typically observed in marine sediments
Source: Front Microbiol. 2024 Jul 22;15:1407459. doi: 10.3389/fmicb.2024.1407459 (PMC11298437; doi:10.3389/fmicb.2024.1407459)
Supplement: Supplementary file 1 [file Data_Sheet_1.docx]

Supplementary Material


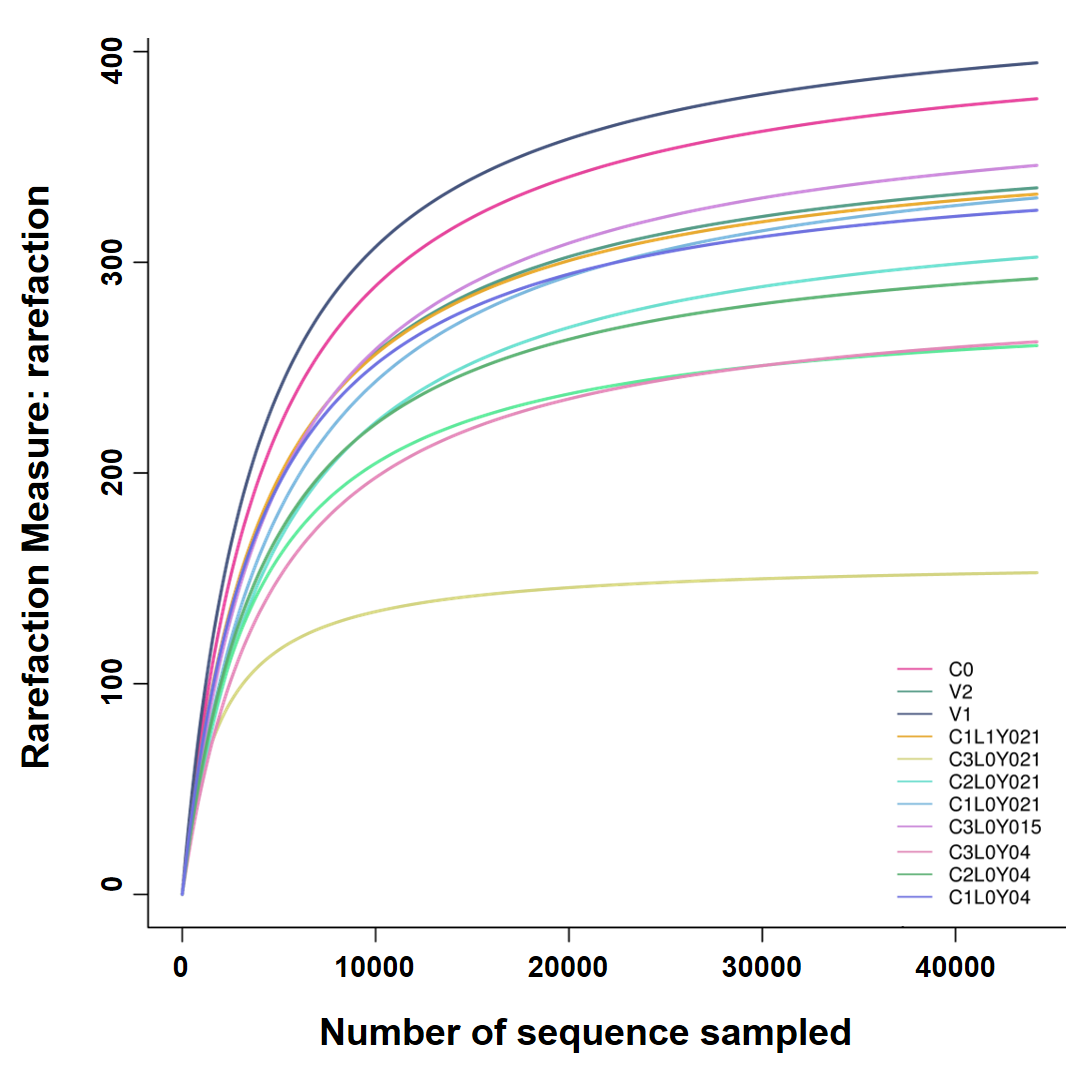


**Supplementary Figure 1.** Rarefaction curves of the 11 samples.


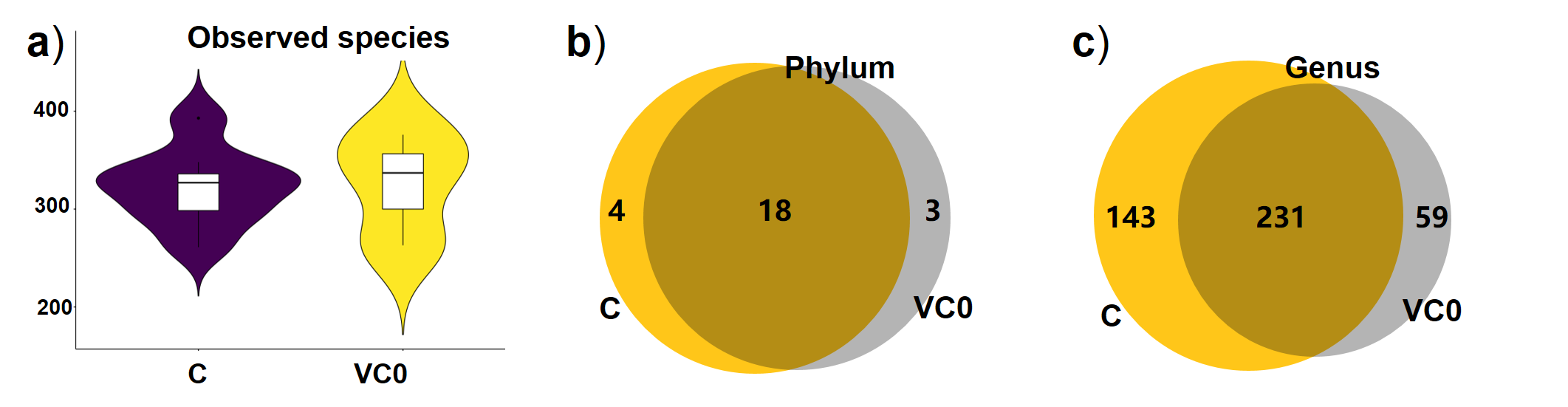


**Supplementary Figure 2.** Violin plot and Venn diagrams of C and VC0 groups. a) Violin plot (median, min and max) showing alpha diversity (the number of observed species) of the two group. The analyses were performed at ASV level. b) and c) Venn diagram showing the numbers of shared and unique bacterial ASVs.


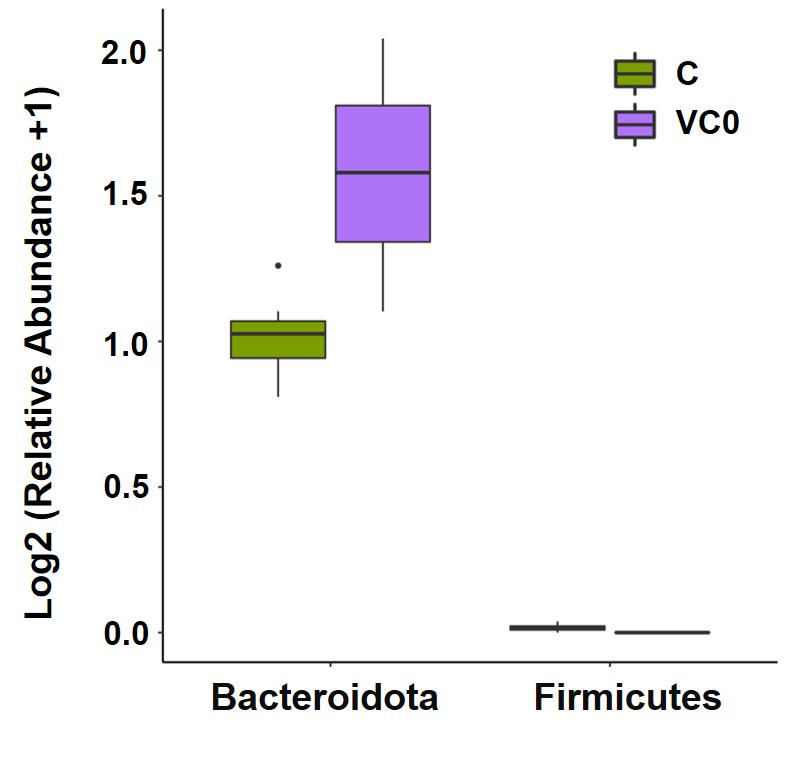


**Supplementary Figure 3.** Bar plot of significantly different bacterial phylum between C and VC0 groups. The Y-axis shows the relative abundance of the bacterial phylum. The analysis were performed at the phylum level for 16S rRNA gene amplicons via QIIME (version 2) plugin.


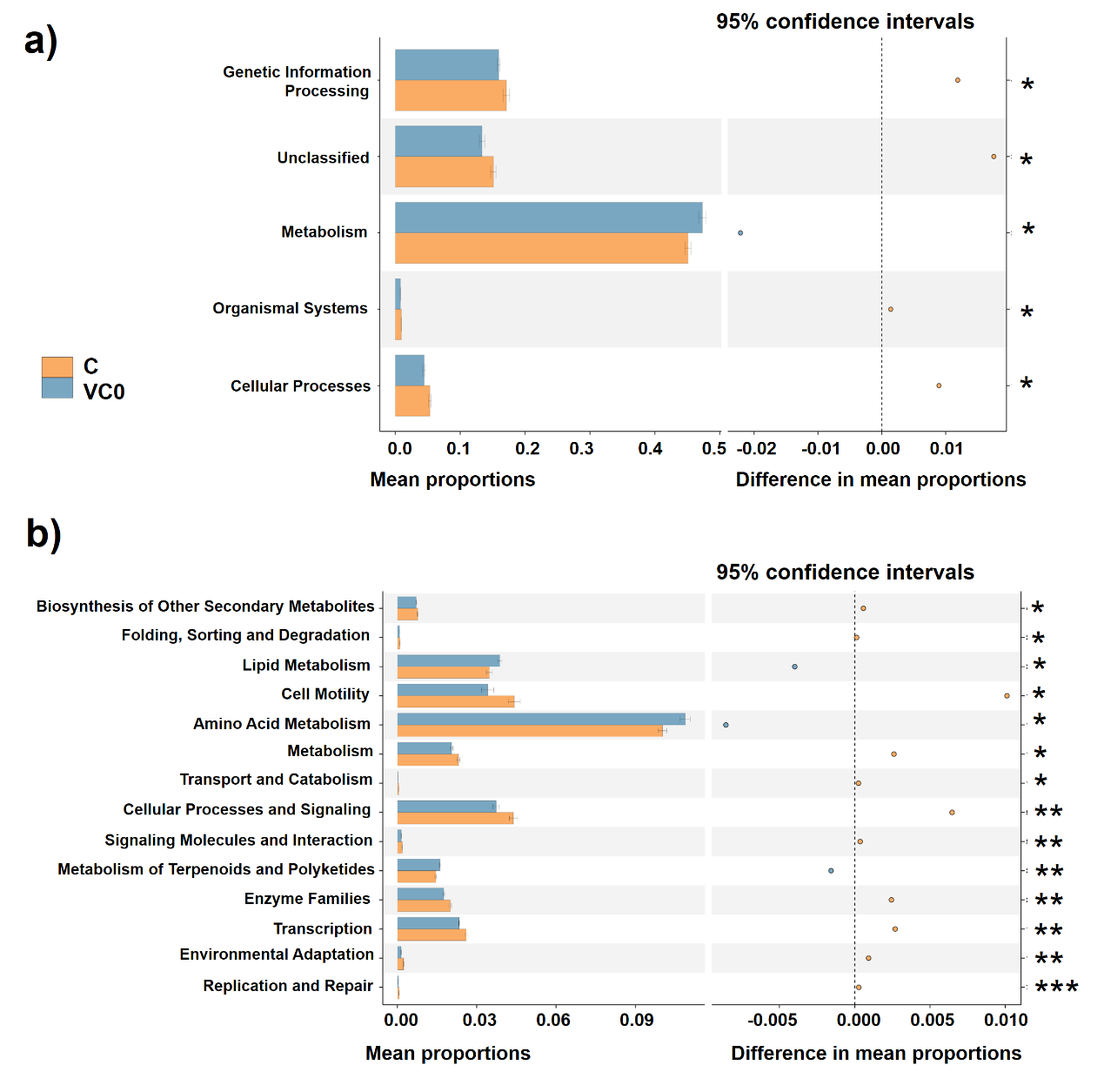


**Supplementary Figure 4**. Prediction of the differential function of bacterial microbiomes between the C (orange) and VC0 (blue haze) groups in KEGG categories. a) KEGG level 1; b) KEGG level 2. Gene functions were predicted from 16S rRNA gene-based microbial compositions at the ASV level using the PICRUSt algorithm to make inferences from KEGG annotated databases via QIIME (version 2) plugin. Relative signal intensity was normalized by the number of the genes for each indicated metabolic pathway. 95% confidence interval is where 95% of the samples fall within this range. The right longitudinal axis are the *p*-values calculated by the *t*-test method. Symbols *, **, and *** indicate the difference at significant levels with *p*<0.05, *p*<0.01, and *p*<0.001, respectively. Mean proportions indicate the mean proportional of the functional term in the two group. Difference in mean proportions is shown as the distance from the dots to the dashed line, representing different values between the relative abundance of a certain functional term in a group and the mean proportions of the certain functional term. The dots of the two groups are on each side of the dashed line, in which group the relative abundance is higher, the dots will be shown as the color of the group.
